# Hybrid cuprous halides enable high-sensitivity luminescence lifetime thermometry with exceptional water resistance

**DOI:** 10.1038/s41377-025-02041-3

**Published:** 2025-10-11

**Authors:** Yimin Yang, Jialiang Xu

**Affiliations:** School of Materials Science and Engineering, Tianjin Key Laboratory of Metal and Molecular Materials Chemistry, Frontiers Science Center for New Organic Matter, Nankai University, Tianjin, 300350 China

**Keywords:** Photonic crystals, Nonlinear optics

## Abstract

A new type of hybrid cuprous halide (TPP_3_Cu_2_Br_2_) is reported for luminescence lifetime thermometry, featuring both extraordinary water stability and ultrahigh temperature sensitivity. This material overcomes the long-standing trade-off between sensitivity and water resistance in metal halide-based thermometers, opening up new avenues for temperature sensing in humid or aqueous environments.

## Abstract (web summary)

TPP_3_Cu_2_Br_2_ enables precise underwater temperature sensing with minimal error, leveraging its excellent stability and unique temperature-dependent luminescence lifetime.

## Graphical abstract

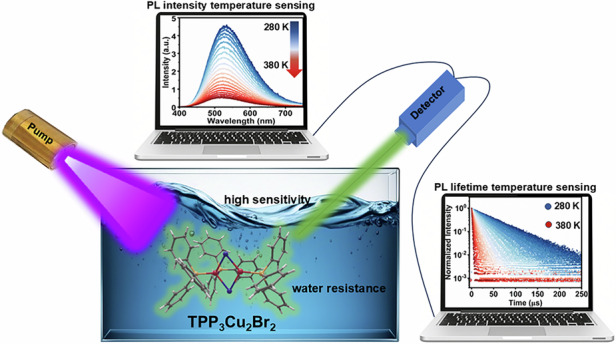

Optical temperature measurement technology, particularly strategies based on monitoring changes in optical parameters (e.g., photoluminescence (PL) intensity or lifetime) with temperature, has been widely applied in temperature sensing^1–5^. However, traditional methods (e.g., infrared thermal imagers), which primarily rely on PL intensity measurements, are often susceptible to various interfering factors. These factors include, but are not limited to: fluctuations in the excitation light source, variations in sample concentration, the photobleaching effect, sample scattering properties, and wavelength-dependent absorption differences. These interference sources may cause significant errors in the temperature measurement methods based on PL intensity in practical applications^6–9^.

In contrast, PL lifetime-based optical temperature measurement technology demonstrates significant advantages. Since the PL lifetime essentially reflects the duration of the excited state of the luminescent center, it primarily depends on the intrinsic properties of the material and its local environment (e.g., temperature), and is relatively insensitive to external factors affecting PL intensity (e.g., light source fluctuations, concentration changes). Therefore, PL lifetime-based methods can fundamentally overcome the inherent limitations of traditional intensity-dependent temperature sensing methods (e.g., infrared thermal imaging)^10^.

At present, PL lifetime-based temperature sensing materials primarily focus on two types of doped phosphors: Ln^3+^-ion-doped and ns^2^-ion-doped materials. For Ln^3+^-doped materials (e.g., europium, terbium, and other rare earth ions), their luminescence typically originates from 4f-4f transitions. Due to effective shielding by the outer 5s^2^5p^6^ electron shell, these transitions are less influenced by the surrounding crystal field environment, resulting in an insufficiently large variation range (Δτ) of PL lifetime with temperature, which in turn limits their temperature sensing sensitivity (S_r_)^11–15^. On the other hand, ns^2^-ion-doped materials (e.g., Te^4+^, Bi^3+^, Sb^3+^, Sn^2+^) typically exhibit higher temperature sensitivity (i.e., larger Δτ), but their PL lifetime is often very short (nanosecond (ns) level). Such short lifetimes require complex and expensive ultrafast time-resolved detection systems (e.g., time-correlated single photon counting) and short-pulse lasers, which significantly increase system complexity and cost, limiting their widespread application in most practical scenarios^16–18^. Therefore, the development of new temperature-sensing materials that combine high temperature sensitivity (i.e., a large PL lifetime change rate) and long lifespan (for easy detection) has become an urgent need in this field.

Over the past decade, metal halides (MHs), as emerging materials, have gained extensive attention in PL lifetime-based temperature sensing^19–24^. Among them, organic-inorganic hybrid metal halides (OIMHs) are particularly notable owing to their significant temperature-dependent structural characteristics. Upon heating, the thermal motion of organic cations (or molecules) in OIMHs intensifies, often leading to severe lattice expansion (e.g., phase transitions, structural distortions). This lattice change induces defect states, alters exciton dynamics, or affects electron-phonon coupling strength, thereby significantly modulating the radiative recombination rate of self-trapped excitons (STEs) in the material, ultimately resulting in a strong temperature dependence of PL lifetime. OIMHs typically exhibit relatively long PL lifetimes (microsecond (μs) to millisecond (ms) level), making them highly suitable for the development of sensitive and easily measurable PL lifetime-based thermometers^25–28^. However, OIMH-based temperature sensing materials often suffer from their poor water stability. Many OIMHs decompose easily in humid environments or water, which severely limits their practical application in scenarios requiring water contact or high humidity (e.g., biomedicine, environmental monitoring). Despite significant progress in OIMH research, developing OIMHs with both excellent water stability and high temperature sensitivity in PL lifetime-based temperature measurement remains a major challenge^29–31^.

A potentially effective strategy to overcome the water stability challenge is the introduction of hydrophobic organic molecules into OIMH structures. In a recent study published in *Light: Science & Applications*, Xueyuan Chen, Datao Tu, Luping Wang et al. addressed this challenge by developing a zero-dimensional hybrid cuprous halide, TPP_3_Cu_2_Br_2_ (TPP = triphenylphosphine), which simultaneously achieves ultrahigh temperature sensitivity and exceptional water resistance^32^. This breakthrough stems from the unique design integrating hydrophobic organic TPP molecules with [Cu_2_Br_2_] dimers, creating a material that sets new performance benchmarks for MH-based thermometers (Fig. 1).

**Fig. 1:**
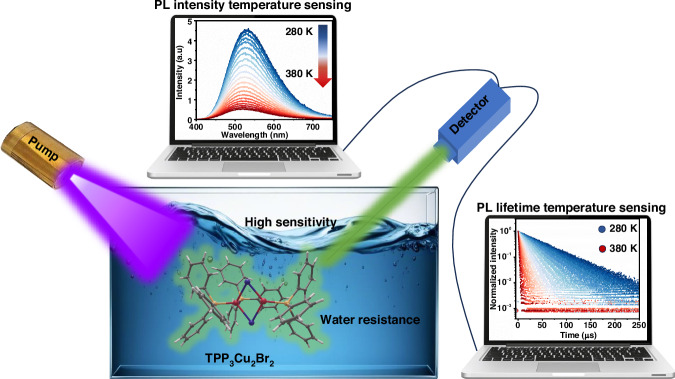
**Schematic diagram of water environment temperature sensing.** Underwater temperature detection is achieved by exciting TPP_3_Cu_2_Br_2_ in water with 355 nm pump light to generate 524 nm green light. Benefiting from the excellent underwater stability and unique temperature-dependent luminescence lifetime of TPP_3_Cu_2_Br_2_, in the field of underwater temperature sensing, the lifetime-based TPP_3_Cu_2_Br_2_ material demonstrates extremely low error. This fully proves the reliability of lifetime-sensing technology in practical applications. Measured images reproduced from ref.^32^

The key feature of TPP_3_Cu_2_Br_2_ is its extraordinary temperature-dependent luminescence lifetime. Benefiting from a TPP-induced soft lattice, the material exhibits giant thermal expansion (3.6% volume increase from 300 to 380 K), far exceeding that of typical MHs (<0.5% expansion). This expansion drives significant lattice distortion, causing the STE luminescence lifetime to plummet from 51.2 μs (280 K) to 0.97 μs (380 K), retaining only 1.9% of its initial value. This dramatic change corresponds to a maximum sensitivity (S_r_) of 12.82% K^−1^, among the highest for undoped MHs. Unlike conventional MHs that degrade rapidly in water, TPP_3_Cu_2_Br_2_ retains 97.3% of its luminescence intensity after 15 days of immersion. This resilience stems from hydrophobic TPP molecules forming a protective barrier. Their large steric hindrance and rigid conformation prevent water molecules from accessing the hydrophilic [Cu_2_Br_2_] dimers. Additionally, strong Cu-P covalent bonds in the lattice resist water-induced ionization, further enhancing structural stability. When used for underwater temperature sensing, TPP_3_Cu_2_Br_2_ (lifetime-based) exhibits minimal error (1.09 °C at 10 mm depth), outperforming intensity-based methods that suffer from increased light scattering and absorption at greater depths. This underscores the robustness of lifetime-based sensing in real-world applications. The integration of ultrahigh sensitivity and water resistance in TPP_3_Cu_2_Br_2_ represents a pivotal advance in MH-based thermometry. By leveraging the synergistic effect of organic-inorganic hybridization, Chen et al. have not only resolved the long-standing stability-sensitivity dilemma but also expanded the application scope of luminescent thermometers to wet environments—from monitoring microzone temperatures in biological tissues to underwater industrial sensing.

Looking ahead, this work paves the way for exploring hybrid metal halides with tailored organic components, potentially unlocking even higher performance. As researchers refine these materials, we can anticipate a new generation of compact, robust, and highly sensitive thermometers that redefine possibilities in both scientific research and daily life.
